# Latent profile analysis and related factors for fear of progression among nasopharyngeal carcinoma patients

**DOI:** 10.3389/fpsyg.2026.1875992

**Published:** 2026-09-18

**Authors:** Mimi Zheng, Shuman Wang, Yu Zhu, Hongwei Wan

**Affiliations:** 1 Department of Nursing, Shanghai Proton and Heavy Ion Center, Fudan University Cancer Hospital, Shanghai, China; 2 Shanghai Key Laboratory of Radiation Oncology, Shanghai, China; 3 Shanghai Engineering Research Center of Proton and Heavy Ion Radiation Therapy, Shanghai, China

**Keywords:** cross-sectional study, fear of progression, latent profile analysis, nasopharyngeal carcinoma, related factors

## Abstract

**Background:**

Fear of progression is a common psychological burden among nasopharyngeal carcinoma patients, persisting for many years following treatment completion. However, most existing studies focus on total scale scores and overlook the potential heterogeneity in profiles of fear of progression among nasopharyngeal carcinoma patients. Consequently, this study aims to identify the latent profiles of fear of progression among nasopharyngeal carcinoma patients and to explore factors of these profiles, including sociodemographic characteristics, clinical characteristics, illness perception, and meaning in life.

**Methods:**

A total of 296 nasopharyngeal carcinoma patients completed the sociodemographic and disease related questionnaire, fear of progression questionnaire-short form, brief illness perception questionnaire and purpose in life test. Latent profile analysis was used to explore the subgroups of fear of progression. The variables associated fear of progression sub-profiles were compared using chi-square test, Fisher's exact test, Kruskal–Wallis H test and multivariate logistic regression analysis.

**Findings:**

The fear of progression among nasopharyngeal carcinoma patients can be categorized into three profiles: low, moderate, and high fear profiles. The results of the multivariate logistic regression showed that gender, age and initial diagnosis/recurrence were signifcant factors associated with the subgroups of fear of progression. Female patients, patients aged 18–30 years, and those with recurrent nasopharyngeal carcinoma (NPC) were prone to high fear profile. The result indicated that the higher illness perception and lower meaning in life, the greater the likelihood of being classified into a higher fear profile.

**Implications:**

Three latent profiles of fear of progression exist in the nasopharyngeal carcinoma patients. Healthcare providers should develop and implement tailored strategies based on the characteristics of three profiles to alleviate fear of progression and maintain mental health.

## 1 Introduction

Globally, approximately 119,750 new cases of nasopharyngeal carcinoma (NPC) are diagnosed in 2024 (Sung et al., 2026). Markedly higher incidence rates are observed in southern-Chinese provinces including Guangdong, Guangxi, and Hunan, with China contributing approximately 46.9% of the worldwide NPC disease burden (Zhang et al., 2024). Radiotherapy constitutes the primary treatment modality for NPC (Bauer et al., 2025). However, locoregional recurrence at the nasopharyngeal site and/or cervical lymph nodes, as well as distant metastasis, occur in 20%−30% of patients, with 65%−85% of recurrences manifesting within 3 years (Bauer et al., 2025; Wang C. et al., 2025; Wang S. et al., 2025). Recurrence greatly increases treatment difficulty and raises the risk of serious complications, such as radiation-induced encephalopathy and fatal hemorrhage, which greatly impacts patient survival rates and quality of life due to the complexity of re-diagnosis and the toxicity of re-irradiation (Huang et al., 2023; Gordon et al., 2023). The high recurrence incidence of nasopharyngeal cancer, combined with increasing retreatment complexity and reduced survival probabilities, inspires substantial fear among NPC patients.

Fear of progression (FoP) is defined as an individual's fear response to disease-related realities, specifically the fear of various biopsychosocial consequences arising from disease progression or the fear of disease recurrence (Herschbach and Dinkel, 2014). FoP represents one of the most prevalent psychological concerns among cancer survivors (Smith et al., 2022; Vandraas et al., 2021), with 22%−87% reporting moderate-to-severe FoP and up to 15% experiencing severe FoP (Boide et al., 2025; Simard et al., 2013). Lin et al. (2026) found that patients newly diagnosed with stage III or higher non-metastatic NPC experienced moderate FoP at 3, 6, and 12 months following the completion of radiotherapy. Even 5 years after being released from the hospital, up to 29% of patients still experience FOP (Qiu and Yang, 2020). Current evidence suggests that FoP gives rise to uintrusive, uncontrollable obsessive thoughts (Simard et al., 2010), induces distressing symptoms such as pain, fatigue and sleep disturbances (Dupont et al., 2014), prompt over-testing (Sarkar et al., 2015; Takeuchi et al., 2020), and substantially increases family-related financial burdens (Mirosevic et al., 2019). Furthermore, FoP increases vulnerability to psychological morbidities, such as anxiety, depression, and perceptions of uncertainty (Williams et al., 2021). FoP fuels concerns regarding drug-related side effects, reduces treatment adherence and therapeutic efficacy (Luigjes-Huizer et al., 2022; Tutelman and Heathcote, 2020), and may even increase the risk of suicide (Kim et al., 2020).

Research indicates that FoP is influenced by the interactions of socio-demographic, clinical and psychocognitive variables (Jrad et al., 2024). In the socio-demographic domain, female gender and younger age are often regarded as factors associated with high FoP (Chen et al., 2025), although associations with marital status, educational level, and family income vary across different cultural and healthcare contexts (Sahin et al., 2025). From a clinical perspective, advanced-stage cancer, recurrence of a previous illness, and intolerable treatment-related symptoms are observable clinical factors associated with increased severity of FoP, reflecting the psychological impact of objective disease-related threats (Calderon et al., 2024; Wang et al., 2022). The cognitive-emotional dimension also exerts a significant influence: negative evaluations of the disease, low tolerance for uncertainty, and accompanying symptoms of anxiety and depression are all consistently associated with heightened FoP (Wang et al., 2022; Shen et al., 2024; Fenech et al., 2024). In addition, psychological resources such as social support may alleviate emotional distress (Zhang et al., 2025). The extent of the influence of these factors may vary depending on tumor type and sociocultural context, highlighting the value of conducting FoP research across diverse patient populations (Alimolk et al., 2024; Hampton et al., 2024). Although abundant literature has described FoP among heterogeneous cancer populations, FoP research specifically targeting NPC patients remains limited. Considering the unique epidemiological characteristics of NPC and the psychological impairment attributed to FoP, clinical attention should be directed toward FoP among NPC survivors.

Additionally, this study was guided by the Common-Sense Model of Self-Regulation (CSM) (Fardell et al., 2016) and the meaning-making model (Park, 2010). The CSM focuses on illness perceptions, which describe patients' cognitive-affective representations of disease. Illness perceptions captures patients' subjective understanding of cancer severity, timeline, consequences, and treatment burden, which constitute the primary threat appraisal of cancer stressors. Cai et al. (2026) investigated 243 cancer patients with gastrointestinal cancer and confirmed illness perception has directly positive effect on FoP. The meaning-making model suggests that meaning-making serves as an adaptive resource for individuals confronting life-threatening disease. Krok et al. (2024) including 246 adult patients with breast cancer demonstrated that poorer meaning-making was directly associated with higher FoP in a serial-mediation study. Research suggests that meaning-making and fear of recurrence may be the underlying mechanisms behind the association between psychological flexibility, self-esteem, and total pain in cancer patients following treatment (Krok et al., 2024). Accordingly, we hypothesize that FoP classification is influenced by both illness perception and the meaning in life, rather than being determined by any single variable. Although the role of mediation of meaning in life and FoP have been established, few studies have adopted latent profile analysis to explore how illness perception and meaning in life together characterize distinct FoP subgroups among NPC patients.

Latent profile analysis (LPA) is an individual-centered approach that utilizes discrete latent profiles to capture associations among external indicators while preserving their local independence (Ferguson et al., 2019). LPA provides additional information that fail to obtain from total scores or clinical cut-offs. The total score reflects only the overall severity of FoP and overlooks the heterogeneity among patients with similar total scores. In contrast, LPA capture the psychological characteristics of each profile. The LPA can identify the heterogeneity of FoP subgroups and facilitate more targeted psychological assessment and intervention. We propose the following hypotheses: (a) FoP of NPC patients can be divided into distinct latent profiles; (b) each profile exhibits specific demographic and clinical characteristics; and (c) latent profiles of FoP among NPC patients are associated with illness perception and meaning in life. Supplementary sub-hypotheses for hypothesis (c) are as follows: First, patients characterized by high illness perceptions coupled with low meaning in life will demonstrate high FoP across all scale items. Second, patients with low illness perceptions and high meaning in life will present persistently low FoP. Third, patients reported high illness perception and high meaning in life will contribute moderate FoP.

## 2 Methods

### 2.1 Study design

The cross-sectional study was approved by the Ethics Committee of the Shanghai Proton and Heavy Ion Center (Ethics Approval Number: B2202-53-02). This study was reported in accordance with the Strengthening the Reporting of Observational Studies in Epidemiology (STROBE) statement (Dewidar et al., 2025).

### 2.2 Participants

From February 2022 to June 2023, patients diagnosed with nasopharyngeal carcinoma and admitted to the Shanghai Proton and Heavy Ion Center were recruited as study participants. The inclusion criteria were: (1) clinical diagnosis of NPC; (2) age ≥ 18 years; and (3) provision of written informed consent. The exclusion criteria were as follows: (1) a history of mental illness or use of specific psychiatric medications; (2) receipt of psychological therapy during the study period; (3) sudden exacerbation of the medical condition; and (4) cognitive impairments and/or communication difficulties. The sample size was calculated by the following formula (Pan et al., 2024).


$$\begin{array}{l} \text{n}={\text{Z}}_{1-\text{α}{/}_{2}}^{2}\times \text{p}\left(1-\text{p}\right)/{\text{d}}^{2} \end{array}$$ (1)

The confidence level was 95% (two-sided), $Z_{1-\alpha /2}$(standard normal variate) was 1.96, *p* represented the estimated FoP prevalence (69.2%) among NPC patients (Yang et al., 2023), and *d* was 6% for the margin of error. Thus, the calculated minimum required sample size was 227. Accounting for 20% anticipated proportion of invalid or excluded questionnaires, the final target sample size was set to 272 participants.

### 2.3 Data collection

Prior to questionnaire administration, trained research staff distributed paper questionnaires and written informed-consent forms. In addition, patients were assured that all collected data would be kept confidential. Questions raised during the completion of the questionnaires were addressed promptly. Clinical data were collected through manual review of electronic medical records. Questionnaires were reviewed, verified, and retrieved immediately on-site by the researchers. Questionnaires exhibiting patterned responses or identical answers across all items were considered invalid and subsequently excluded.

### 2.4 Measures

#### 2.4.1 Sociodemographic and clinical questionnaire

The sociodemographic questionnaire collected information: age, gender, education level, marital status, whether they have children, and yearly income. Disease-related characteristics included initial diagnosis/recurrence, tumor stage, and disease duration.

#### 2.4.2 Fear of progression questionnaire-short form (FoP-Q-SF)

The Fear of Progression Questionnaire-Short Form (FoP-Q-SF) was developed by Mehnert et al. (2006). The Chinese version of the FoP-Q-SF was translated and culturally adapted by Wu et al. (2015). The scale has 12 items over two dimensions, including physical health (items 1, 2, 3, 5, 9, and 10) and social/family functioning (items 4, 6, 7, 8, 11, and 12). Each item is rated on a five-point Likert scale. Total scores range from 12 to 60, with higher scores indicating greater FoP. A cut-off score of ≥34 indicates clinically relevant psychological distress and suggests the need for psychological support. Reliability and validity testing conducted in patients with initial diagnosis liver cancer yielded a Cronbach's α coefficient of 0.883 for the overall scale (Wu et al., 2015).

#### 2.4.3 Brief illness perception questionnaire (BIPQ)

The Brief Illness Perception Questionnaire (BIPQ) was used to assess NPC patients' perceptions and feelings regarding the severity of their disease. This instrument was developed by Broadbent et al. (2006). The Chinese version of the BIPQ was translated and culturally adapted by Sun et al. (2015). The scale contains eight items categorized into three dimensions: illness cognition, patient emotion, and disease understanding. Total scores range from 0 to 80, with higher scores indicating lower levels of disease cognition and greater negative emotions related to illness perception. The Cronbach's α coefficient for the Chinese version of the BIPQ was 0.831 (Sun et al., 2015).

### 2.4.4 Purpose in life test

Meaning in life was measured using the Chinese-language version of the Purpose in Life (PIL) test (Song, 1992), which includes four dimensions: purpose in life (seven items), quality of life (five items), value of life (six items), and freedom of life (two items), totaling 20 items. We used a seven-point Likert scale to enhance measurement accuracy and better capture subtle differences across items. With a range of 20-140, higher scores indicate greater meaning. The Cronbach's alpha coefficient for the scale was 0.908 (Wang C. et al., 2025; Wang S. et al., 2025).

### 2.5 Data analysis

Latent profile analysis (LPA) was performed using Mplus version 8.3. The 12 item scores of the FoP-Q-SF were entered as manifest variables, and the number of latent profiles was incrementally increased until optimal model fit was achieved. Model fit was evaluated using the following indices (Ferguson et al., 2019): (1) Akaike information criterion (AIC), Bayesian information criterion (BIC), and adjusted Bayesian Information Criterion (aBIC), with lower values indicating better model fit; (2) entropy, with values greater than 0.80 indicating high classification accuracy; and (3) the Lo–Mendell–Rubin adjusted likelihood ratio test (LMR) and the bootstrap likelihood ratio test (BLRT), where a *p* < 0.05 indicated that a model with *K* classes fit significantly better than a model with *K*−1 classes (Nagin, 2005). Subsequent analyses were conducted using SPSS version 26.0. The identified latent profiles were used as grouping variables for between-group comparisons. Categorical variables were analyzed using the chi-square test or Fisher's exact test, while continuous variables were analyzed using one-way analysis of variance or the Kruskal–Wallis H test. Multinomial logistic regression was applied to identify factors associated with latent profile membership. Statistical significance was set at α = 0.05.

## 3 Results

### 3.1 Participant characteristics

The mean age of the participants was 43.17 years (range: 18–76 years). Among the participants, 60.6% were male, 62.3% held a college degree or higher, 82.5% had children, and 80.5% reported no religious affiliation. In addition, 80.8% were newly diagnosed with NPC, 60.9% had tumor stage II or III, and 76.7% had a disease duration of less than 1 year (see Table 1).

**Table 1:** Demographic and clinical characteristics of three latent profiles (*n* = 296).

Variable	Class 1 (*n* = 71)	Class 2 (*n* = 164)	Class 3 (*n* = 61)	χ2	*P*
Age(year)				53.794	**< 0.001** ^*^
18–30	2(2.8)	21(12.8)	21(34.4)		
31–45	25(35.2)	92(56.1)	32(52.5)		
46–60	31(45.1)	43(26.2)	4(6.6)		
>60	12(16.9)	8(4.9)	4(6.6)		
Gender				25.218	**< 0.001**
Male	59(83.1)	96(58.5)	25(41)		
Female	12(16.9)	68(41.5)	36(31)		
Religion				4.36	0.332^*^
None	56(78.9)	138(84.1)	45(73.8)		
Buddhism	13(18.3)	22(13.4)	15(24.6)		
other	2(2.8)	4(2.4)	1(1.6)		
Education				8.422	0.202^*^
Junior high school or below	4(5.6)	11(6.7)	4(6.6)		
High school or equivalent	19(26.8)	47(28.7)	26(42.6)		
College	36(50.7)	92(56.1)	27(44.3)		
Postgraduate and above	12(16.9)	14(8.5)	4(6.6)		
Have any children				2.179	0.354
Yes	61(85.9)	131(79.9)	53(86.9)		
No	10(14.1)	33(20.1)	8(13.1)		
Yearly income				4.676	0.322
< 150,000/year	22(31)	59(36)	23(37.7)		
150,000–300,000/year	30(42.3)	49(29.9)	23(37.7)		
≥300,000/year	19(26.8)	56(34.1)	15(24.6)		
Initial diagnosis/recurrence				15.08	**0.001**
Initial diagnosis	68(95.8)	129(78.7)	43(70.5)		
Recurrence	3(4.2)	35(21.4)	18(29.5)		
Disease duration (month)				7.835	0.25
≤ 3	24(33.8)	49(29.9)	19(31.1)		
3–6	19(26.8)	55(33.5)	20(32.8)		
6–12	15(21.1)	22(13.4)	4(6.6)		
≥12	13(18.3)	38(23.2)	18(29.5)		
Cancer stage				2.961	0.818
Stage I	12(16.9)	23(14)	10(16.4)		
Stage II	24(33.8)	47(28.7)	16(26.2)		
Stage III	17(23.9)	56(34.1)	21(34.4)		
Stage IV	18(25.4)	38(23.2)	14(23)		

^*^Fisher's exact test. Bolded *p*-values indicate statistical significance at *p* < 0.05.

### 3.2 Results of latent profile analyses

In total, 296 NPC patients were included in the final analysis, the mean total score on the FoP-Q-SF was 30.05 ± 8.41, with mean scores of 16.02 ± 4.48 for the physical health dimension and 14.03 ± 4.58 for the social/family dimension. When increasing the number of latent profiles from one to three, the AIC, BIC, and aBIC values declined, but the LMR and BLRT tests were statistically significant (both *P* < 0.05), and entropy reached its maximum value. When the number of profiles increased from four to six, the LMR and BLRT were no longer statistically significant, and entropy decreased. Following a thorough comparison of the model fit indices, the best-fitting model was selected for three latent profiles (Table 2). And the entropy value of 0.896 indicated high classification accuracy. Figure 1 shows the mean scores of FoP across the three profiles. Profile 1 had an average FoP score of 19.19 ± 0.25, with the lowest mean score across all items. This group was designated the “low fear” profile (*n* = 71, 24%). Profile 2 demonstrated intermediate item-level mean scores, with an average FoP score of 29.98 ± 0.29, and was named the “moderate fear” profile (*n* = 164, 55.4%). Profile 3 with the highest item mean score and average FoP score of 40.37 ± 0.46 was labeled the “high fear” profile (*n* = 61, 20.6%).

**Table 2:** Latent profile analysis model fit statistics.

Model	AIC	BIC	aBIC	Entropy	LMRT (*P*)	BLRT (*P*)	Probability of class
1	10,248.994	10,337.562	10,261.451				1
2	9,377.781	9,514.324	9,396.985	0.85	0.0146	0.0153	0.48/0.52
3	9,016.541	9,201.059	9,042.493	0.896	0.0002	0.0002	0.24/0.54/0.21
4	8,967.519	9,200.012	9,000.219	0.842	0.3748	0.3804	0.15/0.44/0.24/0.18
5	8,911.816	9,192.283	8,951.263	0.815	0.3708	0.3747	0.23/0.16/0.25/0.21/0.16
6	8,873.392	9,201.843	8,919.587	0.850	0.4922	0.4940	0.15/0.22/0.12/0.32/0.09/0.10

AIC, akaike information criterion; BIC, Bayesian information criterion; aBIC, adjusted Bayesian information criterion; LMR (P), P value for the Lo–Mendell–Rubin; BLRT (P), P value for the bootstrapped likelihood ratio test.

**Figure 1:**
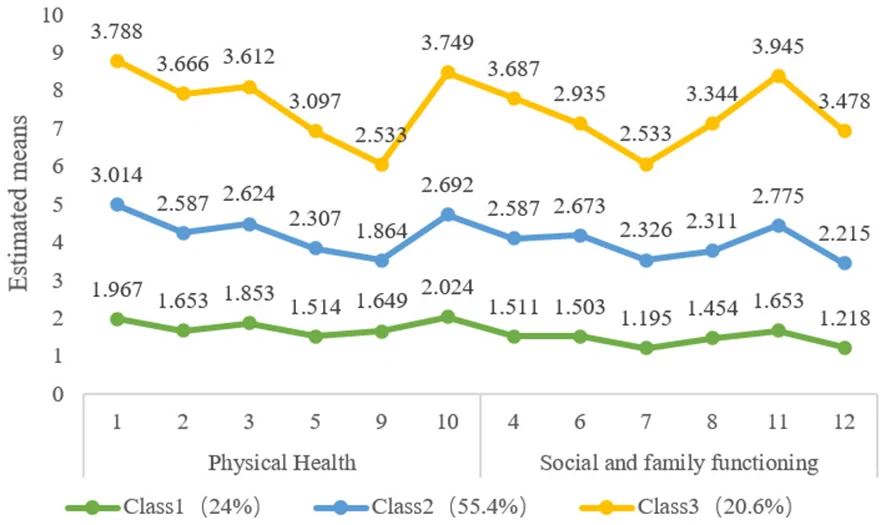
Characteristics of three latent profiles of FOP in nasopharyngeal carcinoma patients.

### 3.3 Univariate analysis of latent profile categories

The sociodemographic and clinical characteristics of the FoP profiles were compared using the chi-square test and Fisher's exact test, respectively. The Kruskal–Wallis H test was used to compare total scores of FoP including physical health dimension and social and family dimension, BIPQ scores, and meaning in life scores among the three profiles (see Tables 1, 3). Significant differences were observed among the three latent profile categories in terms of total FoP scores, the physical health dimension, the social and family functioning dimension, illness perception scores, and meaning in life scores.

**Table 3:** Classification of latent profiles of the scores of the three scales [*n* = 296, mean ± SD/M (P25, P75)].

Variables	All samples (*n* = 296)	Class 1	Class 2	Class 3	*H*	*p* value
FoP	30(24.25, 35)	19(17, 21)	31(28, 33)	41(39, 44)	237.496	**< 0.001**
Physical Health	16(13, 19)	11(9, 12)	16(14, 18)	21(20, 24)	208.008	**< 0.001**
Social and family functioning	14(10, 17)	9(7, 10)	13(14, 16)	18(20, 21)	214.984	**< 0.001**
Illness perception	41.15(36, 46.5)	37.4(30, 43)	40.95(36.85, 45.95)	46.5(40.6, 52.9)	43.047	**< 0.001**
Meaning of life	88(83, 92)	89(86, 95)	88.5(84, 92)	83(78, 88)	28.662	**< 0.001**

FoP fear of progression, Class 1 low fear profile, Class 2 moderate fear profile, Class 3 high fear profile.

### 3.4 Multivariate logistic regression analysis of latent profile categories

Three latent FoP profiles were treated as the multinomial dependent variable, we conducted multivariate logistic regression with sociodemographic characteristics, clinical characteristics, illness perception, and meaning in life as independent variables (Table 4). Compared with the high fear profile, patients in the low fear profile were more likely to be male and initially diagnosed. In addition, female patients, patients aged 18–30 years, and those with recurrent NPC were assigned to high fear profile. Compared with low fear profile, patients in moderate fear profile were more likely to be female, aged 18–30 or 31–45 years. Male patients aged 46–60 years with initial-diagnosed NPC showed higher odds of belonging to the low-fear profile. Higher BIPQ scores (indicating more negative illness perceptions) were associated with higher odds of membership in more severe FoP profiles, whereas higher scores of meaning in life were associated with reduced odds of membership in severe FoP profiles.

**Table 4:** Results of multivariate logistic regression analysis (*n* = 296).

Comparison group	Variables	β	SE	Waldχ^2^	*P*	OR	95%CI	
C1 (ref: C3)	Gender (ref: Female)							
	Male	1.695	0.5	11.483	**0.001**	5.449	2.044	14.526
	Age (year) (ref: >60)							
	18–30	−3.267	1.106	8.728	**0.003**	0.038	0.004	0.333
	31–45	−1.246	0.835	2.229	0.135	0.288	0.056	1.477
	46–60	1.633	0.968	2.842	0.092	5.117	0.767	34.141
	Initial diagnosis/recurrence (ref: recurrence)							
	Initial diagnosis	2.346	0.748	9.827	**0.002**	10.442	2.409	45.266
	Meaning of life	0.138	0.034	16.605	**< 0.001**	1.148	1.074	1.226
	Illness perception	−0.174	0.032	29.012	**< 0.001**	0.84	0.788	0.895
C2 (ref: C3)	Gender (ref: Female)							
	Male	0.579	0.355	2.667	0.102	1.785	0.891	3.577
	Age (year) (ref: >60)							
	18–30	−0.802	0.79	1.032	0.31	0.448	0.095	2.108
	31–45	0.349	0.739	0.222	0.637	1.417	0.333	6.036
	46–60	1.854	0.893	4.311	**0.038**	6.384	1.109	36.738
	Initial diagnosis/recurrence (ref: recurrence)							
	Initial diagnosis	0.611	0.403	2.305	0.129	1.843	0.837	4.057
	Meaning of life	0.075	0.025	8.693	**0.003**	1.078	1.025	1.133
	Illness perception	−0.107	0.026	17.765	**< 0.001**	0.898	0.854	0.944
C2 (ref: C1)	Gender (ref: Female)							
	Male	−1.116	0.39	8.204	**0.004**	0.328	0.153	0.703
	Age (year) (ref: >60)							
	18–30	2.465	0.911	7.318	**0.007**	11.761	1.972	70.148
	31–45	1.595	0.57	7.841	**0.005**	4.928	1.614	15.05
	46–60	0.221	0.58	0.146	0.703	1.248	0.4	3.888
	Initial diagnosis/recurrence (ref: recurrence)							
	Initial diagnosis	−1.735	0.661	6.883	**0.009**	0.176	0.048	0.645
	Meaning of life	−0.063	0.025	6.436	**0.011**	0.939	0.894	0.986
	Illness perception	0.067	0.022	9.466	**0.002**	1.069	1.025	1.116

Class 1 low fear profile, Class 2 moderate fear profile, Class 3 high fear profile.

## 4 Discussion

To identify three latent profiles of FoP among NPC patients, this study investigated the relationships between the three latent profiles and sociodemographic and clinical characteristics, illness perception, and meaning in life. The results revealed that, compared with the high fear profile, patients in the low-fear profile were significantly more likely to be male and to have initial diagnosis. Conversely, female patients, young adults aged 18–30 years, and those with recurrent NPC were classified to high-fear profile. Patients belonging to the moderate fear profile tended to be aged 46–60 years. Additionally, meaning in life was significantly related to FoP latent profiles, whereas illness perception also exhibited significant correlations with profile membership.

The present study identified gender as a significant correlate distinguishing different FoP latent profiles. Compared with the high fear profile, male NPC patients were significantly more likely to be classified into the low-fear profile. Jamal et al. (2025) observed generally higher FoP levels among female breast cancer patients, although their findings were limited by a sample consisting of 99.6% female participants. Similarly, Lin et al. (2024) including lung cancer patients confirmed that female represents a significant correlate of elevated FoP. This finding is often attributed to women's generally higher sensitivity in perceiving and expressing emotions. Female patients tend to adopt coping strategies characterized by rumination when faced with illness. Furthermore, women typically bear a greater burden of caregiving responsibilities within the family. In terms of age, this study discovered that young patients aged 18–30 years were more likely to be assigned to the high fear profile, but middle-aged patients aged 46–60 years tended to fall into moderate fear profile. This finding is consistent with Jamal et al. (2025), who reported that every 10-year increment in age was associated with a 41% decrease in the odds of clinically severe FoP. Younger people often experience stronger social role demands, such as fertility concerns, job advancement, and family formation. The cancer experience causes more severe interruptions in their life goals, heightening fears about future uncertainty. Gnall et al. (2024) confirmed that young cancer patients have increased levels of anxiety and FoP after treatment. On contrary, middle-aged patients may be better equipped to cognitively regulate concerns about recurrence due to more stable psychological development and a wider range of life experiences and coping strategies. This finding is partly consistent with reports from global mixed-cancer cohorts, in which female and young age are typically associated with high FoP (Chen et al., 2025). Although the data were collected at a cancer center in Shanghai, this cohort consisted primarily of patients who had traveled from various parts of the country to receive specialist radiotherapy for NPC. Many participants faced practical pressures associated with seeking medical treatment away from home, including separation from their existing family support networks, additional financial burdens associated with travel, and disruption to their work and daily family life. Under traditional Chinese societal expectations regarding family care and intergenerational responsibilities remained particularly pronounced among female and young patients (Xu et al., 2021). Young patients are mostly at a critical stage in establishing their careers and families. Being diagnosed with cancer, combined with the burden of seeking medical treatment in another city, has further intensified their concerns about employment opportunities, family responsibilities, and the functional impairments caused by the long-term side effects of radiation therapy. These family-centered sources of stress differ from the individual-level suffering emphasized in the West (Sun et al., 2026; Zhao et al., 2025).

This study demonstrated that patients with recurrent NPC were more likely to be classified into the high fear profile, whereas newly diagnosed patients were more likely to belong to the low fear profile. This finding has been corroborated by multiple studies. Jamal et al. (2025) reported that breast cancer patients with a history of recurrence had a 4.27-fold increased risk of clinically significant FoP compared to those without recurrence. Patients who have experienced recurrence, having personally endured the cyclical nature of the disease and the arduousness of treatment, develop a stronger sense of uncontrollability regarding cancer, and traumatic memories become reactivated, leading to significantly elevated FoP. Notably, the finding that initial diagnosed patients are more likely to be classified to the low fear profile should be interpreted with caution. This may be because newly-diagnosed patients are not fully aware of adverse effects and risk of recurrence associated with re-treatments such as the concurrent chemoradiotherapy and massive bleeding from the nasopharynx, etc. NPC patients newly diagnosed have not yet experienced the physical and psychological distress associated with re-treatment, such as pain, anxiety, depression and fear. Gnall et al. (2024) suggested that the level of FoP in the early post-treatment period (averaging 2.5 months) may be underestimated. As patients experience and gradually be aware of the after-effects of treatment, this level of FoP may worsen over time.

In the present study, illness perception was significantly negatively correlated with FoP latent profiles among NPC patients: poorer illness perception corresponded to higher odds of membership within the moderate or high fear profiles. According to the CSM, patients' cognitive representations of illness directly influence their emotional responses and coping behaviors. When patients cannot clearly understand the disease process, etiology, or prognosis, their cognitive schemas become vague and disorganized, readily fostering a sense of uncontrollability that evolves into persistent FoP. These findings underscore the importance for clinical healthcare providers not only to convey information during patient communication but also to ensure that information is clear, coherent, and genuinely understood, helping patients construct a coherent illness narrative that reduces uncertainty-induced fear. Notably, the CSM primarily accounts for the formation of initial threat appraisal. It cannot fully explain why patients with comparable negative illness perceptions exhibit marked heterogeneity in FoP severity, which necessitates complementary insights from the meaning-making model.

As an adaptive resource proposed by the Meaning-Making Model, meaning in life does not eliminate disease-related threats but rather moderates the intensity of emotional distress triggered by illness perceptions. These findings indicate meaning in life is positively correlated with FoP profiles among NPC patients. NPC patients with stronger meaning in life were more likely to be classified into the low fear profile. Patients with higher scores on meaning in life demonstrate significantly greater acceptance of their cancer than those with lower scores. Park's integrated model suggests that when cancer patients are able to find meaning in their lives, they find it easier to accept their cancer experience (Park, 2010). The model posits that there is a positive correlation between meaning-making and acceptance. In addition, cancer patients suffering from the disease may seek meaning, and when they succeed in creating meaning, they are likely to accept their illness more readily. Yang et al. (2023) examined the role of meaning in life in psychological adaptation among Chinese cancer patients. They reported that meaning in life was directly correlated with FoP and also mediated the relationship between FoP and death anxiety. Meaning in life as a psychological resource potentially helps patients reframe illness-related distress, thereby reducing FoP.

Overall, latent-profile findings among NPC patients support the complementary utility of the two theoretical frameworks. The CSM shapes patients' primary threat appraisal of NPC-related stressors. In contrast, meaning-making-related resources further moderate the intensity of subsequent fear. Collectively, these processes give rise to heterogeneous FoP subgroups among NPC patients.

## 5 Limitations

This study has several notable limitations. First, its cross-sectional design precludes any causal inferences. Longitudinal studies are therefore required to explore temporal changes in latent profile membership, as well as the dynamic interrelationships with illness perception and meaning in life. Second, the sample size was limited for latent profile analysis with 12 manifest indicators, and larger-sample studies are required to replicate the identified profile structure. Third, the use of self-report questionnaires for all variables may have introduced common-method bias, and our convenience sampling from a single center may have introduced selection bias. Future research should incorporate data from multiple sources to mitigate these biases. Fourth, although we identified several factors associated with FoP profiles, unmeasured psychological variables and treatment-related variables may have contributed to residual confounding. Fifth, the identified latent profiles have not been validated using independent external measures, such as clinical interviews or additional psychological assessments. Future studies should incorporate such measures to confirm the validity and clinical utility of the three-profile solution.

## 6 Research implications

This study used latent profile analysis to identify three profiles of FoP among NPC patients: the low fear profile, the moderate fear profile and the high fear profile. Significant factors of profile classification included gender, age, disease status, illness perception, and meaning in life. Specifically, women, patients aged 18–30, and patients with recurrent NPC were more probable to be assigned to the high fear profile. Patients in moderate fear profile were more likely to be female, aged 18–30 or 31–45 years. Men, patients aged 46–60, and patients with initial diagnosis were more commonly assigned to the low fear group. Furthermore, higher illness perception and lower meaning in life were associated with a higher likelihood of being classified into the high fear profile. Healthcare providers should prioritize psychological screening and intervention for high risk groups, implementing targeted interventions to alleviate FoP.

## 7 Clinical implications

In clinical practice, LPA-based subgroup classification facilitates individualized care and psychological intervention compared with total-score-based approaches. The findings may assist healthcare identify and deliver interventions for the high fear profile, including young female patients, those with recurrent disease, patients with poor illness perception, and those with a low meaning in life. Stratified intervention strategies can be implemented for patients in high fear profile. For patients with a low fear profile, psychoeducation and informational support should be provided, with a focus on helping patients develop a coherent understanding of their illness. For the moderate fear profile, group psychological interventions such as supportive expressive group therapy may be employed to facilitate emotional expression and the construction of meaning. In the high fear profile, more specialized, individualized psychological treatments such as cognitive behavioral therapy or meaning-centered psychotherapy should be provided. These findings provide evidence to support interventions targeting FoP among NPC patients, thereby improving overall health-related quality of life for NPC patients.

## Data Availability

The original contributions presented in the study are included in the article/supplementary material, further inquiries can be directed to the corresponding author.
